# Impact of CYP2D6 genotype on opioid use disorder deprescription: an observational prospective study in chronic pain with sex-differences

**DOI:** 10.3389/fphar.2023.1200430

**Published:** 2023-05-31

**Authors:** Javier Muriel, Jordi Barrachina, Guillermo Del Barco, Cristian Carvajal, Mónica Escorial, César Margarit, Pura Ballester, Ana María Peiró

**Affiliations:** ^1^ Pharmacogenetic Unit, Clinical Pharmacology Unit, Alicante Institute for Health and Biomedical Research (ISABIAL), Alicante, Spain; ^2^ Occupational Observatory, University Miguel Hernández, Elche, Spain; ^3^ Pain Unit, Department of Health of Alicante-General Hospital, Alicante, Spain; ^4^ Bioengineering Institute, Toxicology and Environmental Health, University Miguel Hernández, Elche, Spain

**Keywords:** CYP2D6, sex-differences, opioid use disorder, deprescription, chronic pain, pharmacogenetics

## Abstract

**Introduction:** Opioid deprescription is the process of supervised tapering and safe withdrawal when a potentially inappropriate use is detected. This represents a challenge in chronic non-cancer pain (CNCP) patients who may respond differently to the procedure. Our aim was to analyze the potential impact of CYP2D6 phenotypes and sex on the clinical and safety outcomes during an opioid use disorder (OUD) tapering process.

**Methods:** A prospective observational study was conducted on CNCP ambulatory OUD patients (cases, *n* = 138) who underwent a 6-month opioid dose reduction and discontinuation. Pain intensity, relief and quality of life (Visual analogue scale, VAS 0–100 mm), global activity (GAF, 0–100 scores), morphine equivalent daily dose (MEDD), analgesic drugs adverse events (AEs) and opioid withdrawal syndrome (OWS, 0–96 scores) were recorded at basal and final visits. Sex differences and CYP2D6 phenotypes (poor (PM), extensive (EM) and ultrarapid (UM) metabolizers based on CYP2D6*1, *2, *3, *4, *5, *6, *10, *17, *41, 2D6*5, 2D6 × N, 2D6*4 × 2 gene variants) were analyzed.

**Results:** Although CYP2D6-UM consumed three-times less basal MEDD [40 (20–123) mg/day, *p* = 0.04], they showed the highest number of AEs [7 (6–11), *p* = 0.02] and opioid withdrawal symptoms (46 ± 10 scores, *p* = 0.01) after deprescription. This was inversely correlated with their quality of life (r = −0.604, *p* < 0.001). Sex-differences were evidenced with a tendency to a lower analgesic tolerability in females and lower quality of life in men.

**Discussion:** These data support the potential benefits of CYP2D6-guided opioid deprescription, in patients with CNCP when OUD is detected. Further studies are required to understand a sex/gender interaction.

## 1 Introduction

The current international analgesic landscape is characterized by a significant global increase in the use of prescription opioid (Upp and Waljee, 2020; Di Gaudio et al., 2021). In fact, 15.2% of the adult Spanish population admits having used opioid analgesics, at some point in their lives (Spanish Observatory on Drugs and Addictions OEDA, 2021), with observed differences in the use and the presence of any opioid use disorder (OUD) between sexes (McHugh et al., 2018). This problematic opioid use has resulted in formulation of practice-specific guidelines as a mechanism to curb current trend (National Academies of Sciences and and Medicine, 2017). In this context, research shows that patients in severe pain despite use of high-dose opioids may experience significant improvement in pain relief and functioning, when their opioid is tapered to a lower, safer dose (Kahan et al., 2011), improving adherence and reducing drug-seeking behaviors (Becker et al., 2018).

Current evidence suggests potential genetic factors that could be used to predict one’s risk of opioid misuse or a problematic use (Singh et al., 2021), harmful (Muriel et al., 2019) or addictive potential (Linares et al., 2014). There is some evidence suggesting CYP2D6 enzyme, responsible for the metabolism of tramadol, codeine and oxycodone, may be more efficient at ultra-rapid metabolizer (UM) synthesizing endogenous opioids (Zahari and Ismail, 2014), experience quicker and higher systemic levels of the active metabolites and therefore, to require lower analgesic doses (Candiotti et al., 2009). However, UM subjects will be prone to higher mu-opioid-related toxicity and a higher risk of adverse events (AEs) (Lopes et al., 2020). In contrast, CYP2D6 poor metabolizers (PMs) would tend to have lower levels of the active metabolites (Haufroid and Hantson, 2015), which may result in reduced analgesic efficacy (Lötsch et al., 2004; Zahari and Ismail, 2014). This could have special impact for females who generally exhibit a lower opioid tolerability in comparison to males (Planelles et al., 2020), which can be turned into differences in opioid´s clearance (Anderson, 2008). Here, scarce data on the effect of sex on the CYP2D6 activity exist, and except for some data related to menstrual cycle influence (Tamminga et al., 1999), explicit recommendations derived through a validated process have not yet been formulated (He et al., 2015).

In this sense, there is increasing evidence in humans and laboratory animals for sex differences in processes of reward and addictive behavior, withdrawal, craving, and relapse due to psychostimulants and opioids (Becker and Chartoff, 2018). In fact, women are more likely to refer and be diagnosed with acute and chronic pain and to be prescribed these drugs in significantly greater numbers than men (Goetz et al., 2021). Although several reports have documented risk factors for opioid use following treatment discharge, yet few have assessed sex differences in long-term opioid use in chronic non-cancer pain (CNCP) management (Cragg et al., 2017; National Academies of Sciences and and Medicine, 2017; Davis et al., 2021).

The primary goal of the present study was to evaluate the impact of CYP2D6 phenotypes and sex influence on OUD deprescription ambulatory CNCP patients. As a primary hypothesis, it was considered that CYP2D6-UM metabolizers would show a different clinical outcome pattern when compared to the other groups, as would be also observed between sexes.

## 2 Materials and methods

### 2.1 Study design and selection of participants

This manuscript adheres to the applicable STROBE guidelines. This prospective observational pharmacogenetic study followed the current Declaration of Helsinki and European Medicines Agency Guidelines for Good Clinical Practice and was approved by the Ethics Committee of The General University Hospital of Alicante. Written informed consent was obtained from all participants prior to their inclusion in the study.

All the CNCP consecutive patients with confirmed OUD who underwent a 6-month opioid deprescription (cases, *n* = 138) by clinical practice at the Pain Unit (PU, General University Hospital of Alicante, Alicante, Spain) from May 2013 to May 2019 were included under the inclusion criteria prior to deprescription: 1) patients aged 18 years or older; 2) with CNCP and long-term opioid use (>6 months); 3) OUD diagnosis according to diagnostic DSM-5 criteria (American Psychiatric Association, 2013) as confirmed by a psychiatrist; and 4) informed consent granted. All the cases were followed-up prospectively for opioid dose reduction and discontinuation. A control group of 231 participants who had previously participated in observational studies from the same setting which were under opioids for chronic pain and no OUD suspicion (Margarit et al., 2019) was included to explore potential differences in terms of sociodemographic, clinical, pharmacological and CYP2D6 phenotypes in comparison to the cases.

### 2.2 Description procedure

The deprescription program was designed, established and executed according to national and international guidelines (Fernández-Miranda, 2007). OUD was defined as a problematic pattern of opioid use that causes significant impairment or distress according to the criteria in the DSM-5 (American Psychiatric Association, 2013). Here, a monitored opioid rotation to tramadol/buprenorphine together with the tapering process (progressive opioid withdrawal through a rotation with dose-reduction and control of any withdrawal symptoms) was conducted through consecutive clinical visits along 6 months (Muriel et al., 2019; Muriel et al., 2018). Depending on the patients’ clinical status they were fully rotated to buprenorphine/tramadol from their basal prescriptions or stayed on their basal prescriptions but lower doses with tramadol as rescue medication. Basal MEDD was ideally 20%–30% reduced at each clinical visit (follow-up visits (1, 2 weeks, 1 and 3 months) and a final visit at 6 months) starting with the total withdrawal of quick-release opioids. Any precipitated opioid withdrawal symptom was carefully monitoring at each clinical visit. Effectiveness, as primary outcome, was considered when neither OUD nor any aberrant opioid use behavior was observed together with a morphine equivalent daily doses (MEDD) reduction minimum of 30% from basal levels - as a clinically meaningful reduction in dose (Perez et al., 2020) - or opioid discontinuation.

### 2.3 Clinical data collection

Demographic characteristics (age, sex) and clinical variables were collected using validated questionnaires and scales completed at each of the patients’ visit. Pain intensity and relief were measured using the Visual Analogue Scale (VAS) (McCormack et al., 1988). Both VAS scales consist of a 100 mm horizontal line ranging from 0 (lowest) to 100 mm (highest). Similarly, VAS-EuroQol Scale (EQ) was used for quality of life assessment (EuroQol, 1990). Opiate Withdrawal Scale (OWS, 0–96 scores) is a questionnaire composed of 32 common symptoms in opioid withdrawal patients (Bradley et al., 1987) rated using scores of 0 (absent) to 3 (severe). The Global Assessment of Functioning (GAF, 0–100 scores) scale was used to assess patient’s psychological, social, and work activity independently from the activity alterations caused by physical limitations. Higher score meaning a better level of activity and life (Jones et al., 1995).

### 2.4 Drug use and adverse events

Opioid and co-adjuvant medications were strictly prescribed by clinical judgement by the physician without any experimental decision. Use of opioid and non-opioid analgesics, NSAIDs, antidepressants (duloxetine), anxiolytics (benzodiazepines) and neuromodulators (pregabalin and gabapentin) was obtained from EHRs. MEDD were estimated using the available opioids equivalent doses (Pergolizzi et al., 2008) and classified as being low (MEDD<100 mg/day) or high (MEDD≥100 mg/day), given the potential increased dose-dependent side-effects (Chapman et al., 2010; Dunn et al., 2010). In addition, MEDD was calculated and analyzed separately in those patients with use of CYP2D6-mediated opioids (oxycodone, hydrocodone, tapentadol, codeine and tramadol).

To assess the tolerability, a questionnaire with the list of the most frequently occurring AEs (according to the opioids’ summary of product characteristics, including “very common” and “common” listings) (Boiarkina and Potapov, 2014) and a blank field to add any other, was used to record patients’ occurrence of AEs (Barrachina et al., 2021). In addition, all ADRs (Wisher, 2012) were collected and classified using the Medical Dictionary for Regulatory Activities (MedDRA, version 20.0) and the Preferred Terms.

### 2.5 *CYP2D6* genotyping

Approximately 2 mL of saliva was collected in PBS containing tubes. Genomic DNA was extracted using an E.N.Z.A. Forensic DNA Kit (Omega bio-tek), according to the manufacturer’s instructions. Genetic analysis was based in usual PCR-methods following the instructions of the Consortium of Pharmacogenetics (CEIBA) and the pharmacogenomics iberoamerican network (RIBEF) for the analysis of samples. XL-polymerase chain (XL-PCR) analysis was used for identification of duplications and deletions (Dorado et al., 2005). These XL-PCR amplifications were carried out in a Mastercycler 384 (Eppendorf, AG, Hamburg, Germany). After the genotype was stablished, the different variants were converted to an Activity Score (AS), which indicated the enzyme’s activity level (null, reduced, normal, increased) (Gaedigk et al., 2008). Presence of SNP *3, *4, *5 or *6 represents an AS of 0, which means a null enzyme activity. Variants *10, *17 and *41 are associated with an AS of 0.5 and *1, *2 and *35 with an AS of 1, representing reduced and normal enzyme activity levels, respectively. Presence of duplications *1xN, *2xN or *35xN suppose an increased enzyme activity level (AS = 2). According to previous classifications, if the AS resulting from the combination of both alleles was zero, the subject was considered as PM; if ranges from 0.5 to 2 as EM; and above 2 as UM (Naranjo et al., 2016).

### 2.6 Statistical analyses

Based on the observational prospective nature of the study and to the inclusion limited by the low frequency of patients with an OUD, a convenience sample was proposed. As an estimated prevalence of 3.2% of OUD was detected in our setting (Muriel et al., 2018). Out of an average of 915 patients/year who visit our PU, 30 potentially eligible subjects per year were expected. Due to the missing or refusing to participate (almost 20%), approximately 24 patients were expected annually. To complement the analysis, a control group from our previous study was proposed. As the condition/event (OUD) is infrequent (<10% prevalence), a complete series of controls was included to achieve a superior number of controls (ratio 2:1).

Data distribution was analyzed using Kolmogorov-Smirnov normality test. Quantitative parametric data are presented as mean (SD) while median (IQR) was used for non-parametric data and discrete variables. Categorical data are expressed by percentages. Comparisons of continuous data between two groups were conducted using a *t*-test for parametric data, meanwhile for non-parametric, U Mann-Whitney test was used. When analyzing categorical data between two groups, Fischer’s exact test was performed. For the analyses of the three metabolic phenotypes, ANOVA test was performed for parametric continuous data and Kruskal-Wallis for non-parametric. In this case, Chi-square test was used for categorical analyses. *t*-test and/or U Mann-Whitney (for PM vs. EM/UM, EM vs. PM/UM and UM vs. PM/EM) were performed too. Gene by sex interaction was explored by invoking a regression model. All the obtained variables included a separate description and analysis by sex.

The Pearson correlation coefficient (r) and its 95% confidence intervals (CI) were calculated to analyze the correlation between opioid withdrawal and quality of life. Two groups (subjects included between 2013–2015 and 2016–2019) were compared to determine if deprescription outcomes changed over time. The MEDD difference between groups was expressed using the Hodges-Lehmann estimator shift with the 95% CI. In the assumption of missing completely at random, complete case (or available case) analysis was performed. A *p* ≤ 0.05 was considered statistically significant. In all cases, multiple testing was adjusted using Bonferroni correction. All statistical analyses were carried out using R (3.2.0 version) software.

## 3 Results

**FIGURE 1:**
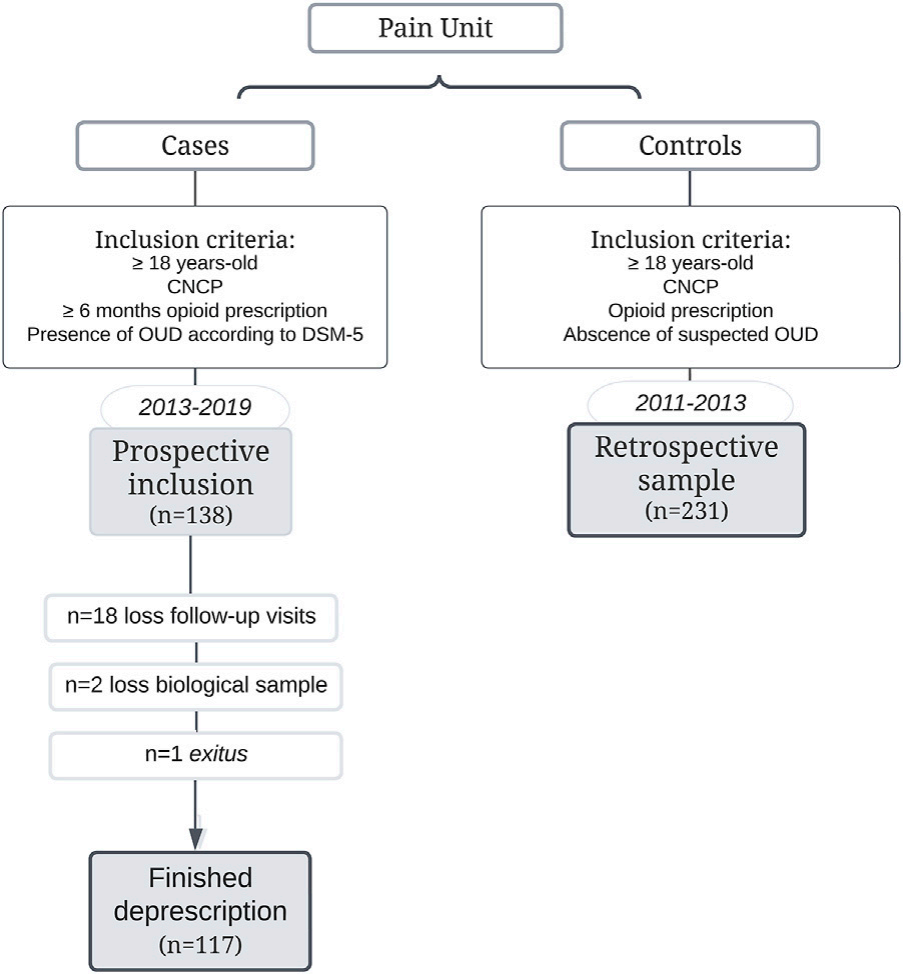
Flow Chart of patients with OUD (cases) along 6 years at Pain Unit and the control group included in the study.

A total of 138 patients (65% female) with an OUD were recruited and enrolled in the ambulatory opioid deprescription. Fifteen percent (*n* = 21) of the patients were lost to follow-up (*n* = 18 did not attend follow-up visits, *n* = 2 no biological samples, and *n* = 1 death due to intestinal pneumatosis) with 117 (66% female), of them completing the program. Data from a total of 231 subjects (64% female) were included as a control group (Figure 1).

At basal visit, cases showed a moderate basal chronic VAS pain intensity (60 (27) mm) and quality of life [45 (24) mm], with mild relief [37 (29) mm] and a mean of [32 (19) OWS scores]. No differences based on the inclusion period or between visits during deprescription were found in these outcomes. Here, patients evidenced “some mild symptoms or difficulties in social, interpersonal relationships or occupational functioning, but generally functioning pretty well” due GAF 71 (15) scores.

Cases were a mean of almost 10 years younger [54 (13) vs. 63 (14) years, *p* < 0.001], with a higher basal pain relief [37 (29) vs. 18 (13) mm, *p* < 0.001] probably due to a higher MEDD [120 (80–200) vs. 40 (0–82) mg/day, *p* < 0.001, difference Hodges-Lehmann: −80; 95% CI of the difference (−90 to −58)] at basal visit (Table 1).

**TABLE 1:** Sociodemographic, clinical, pharmacological and tolerability variables in cases (basal visit) and controls.

Basal visit	Cases (*n* = 138)	Controls (*n* = 231)	*p*-value^†^
Sex (female, %)	65	64	1.00
Age (years, mean (SD))	54 (13)	63 (14)**	**<0.001**
Pain Intensity (VAS, 0–100 mm, mean (SD))	60 (27)	56 (31)	0.09
Pain relief (VAS, 0–100 mm, mean (SD))	37 (29)**	18 **(**13**)**	**<0.001**
Quality of life (EQ, 0–100 mm, mean (SD))	45 (24)	45 (14)	1.00
Total MEDD (mg/day, median (IQR))	120 (80–200)**	40 (0–82)	**<0.001**
AEs (median (IQR))	5 (2–8)*	3 (1–6)	**0.03**
ADRs (%)	13	21	0.07

^†^ Cases vs. controls comparisons using using *t*-test and U Mann-Whitney test for continuous parametric and non-parametric data, respectively, and Fisher’s exact test for categorical data (significant *p* < 0.05 in bold).

*p < 0.05, **p < 0.01 (highest value in bold). VAS, visual analogue scale; EQ, VAS, EuroQol Scale (0–100 mm); AEs, adverse events; ADRs, adverse drug reactions; IQR, interquartile range, expressed in parenthesis as P25 and P75.

### 3.1 Opioid deprescription

Clinical and pharmacological data of the total case population and classified by the CYP2D6 metabolic phenotypes is shown in Table 2.

**TABLE 2:** Demographic and pharmacological variables, in total population and classified by *CYP2D6* metabolic phenotype.

Variables		Cases (basal, *n* = 138; final, *n* = 117)	*CYP2D6* phenotype			*p*-value^†^
			PM (*n* = 7, 6%)	EM (*n* = 98, 85%)	UM (*n* = 10, 9%)	
Age [years, mean (SD)]		54 (13)	47 (12)	54 (13)	59 (14)	0.17
Sex (female, %)		65	71	65	80	0.61
Deprescription Responder (%)		76	80	76	89	0.66
Final opioid use (%)		58	80	55	56	0.55
Total MEDD [mg/day, median (IQR)]	*Basal*	120 (80–200)	120 (60–233)	123 (80–229)	**40 (20–123)** ^+^	0.11
	*Final*	40 (0–120)*	40 (7–65)	40 (0–120)*	80 (0–150)	0.92
*CYP2D6* opioid mediated MEDD [mg/day, median (IQR)]	*Basal*	40 (6–100)	40 (6–100)	40 (6–100)	40 (6–100)	0.81
	*Final*	20 (0–43)**	20 (0–43)	20 (0–43)**	20 (0–43)	0.64
High MEDD (>100 mg/day) (%)	*Basal*	55	60	59	22	0.10
	*Final*	**27** **	0	30**	33	0.33
Pain Intensity [VAS, 0–100 mm, mean (SD)]	*Basal*	60 (27)	63 (22)	61 (27)	62 (29)	0.96
	*Final*	59 (27)	47 (6)	58 (29)	62 (21)	0.44
Pain Relief [VAS, 0–100 mm, mean (SD)]	*Basal*	37 (29)	28 (31)	36 (30)	42 (31)	0.62
	*Final*	40 (28)	57 (21)	41 (30)	39 (22)	0.53
Quality of life [EQ, 0–100 mm, mean (SD)]	*Basal*	45 (24)	38 (26)	46 (25)	46 (21)	0.73
	*Final*	43 (22)	52 (8)	43 (23)	36 (12)	0.46
Opioid Withdrawal [OWS, 0–96 score, mean (SD)]	*Basal*	32 (19)	35 (25)	32 (18)	33 (29)	0.91
	*Final*	32 (20)	10 (0)	30 (20)	**46 (10)** ^**+**^	**0.03**
Global Functionality [GAF, 0–100 score, mean (SD)]	*Basal*	71 (15)	74 (17)	70 (14)	80 (21)	0.48
	*Final*	69 (16)	90 (0)	69 (16)	69 (13)	0.40
Use of non-opioid adjuvants (%)						
Neuromodulators	*Basal*	48	50	52	**0** ^**+**^	0.05
	*Final*	49	40	49	**11** ^**+**^	0.09
Duloxetine	*Basal*	18	33	22	17	0.53
	*Final*	23	20	25	11	0.91
NSAIDs	*Basal*	8	0	7	0	0.63
	*Final*	5	0	4	0	0.52
Simple analgesics	*Basal*	25	17	27	33	0.80
	*Final*	13	40	12	0	0.09
Benzodiazepines	*Basal*	36	17	40	33	0.52
	*Final*	37	20	38	22	0.85

^†^ Comparisons between PM, vs. EM, vs. UM, were performed using ANOVA, or Kruskal-Wallis test for continuous parametric and non-parametric data, respectively and Chi-square test for categorical data.

+ *p < 0.05, **p < 0.01 basal vs. final (lowest value in bold) using *t*-test or U Mann-Whitney test for parametric and non-parametric data, respectively*p* < 0.05 UM vs. PM/EM (UM, value in bold and shaded in grey) using *t*-test or Fisher’s exact test for continuous or categorical data, respectively. PM, poor metabolizer; EM, extensive metabolizer; UM, ultrarapid metabolizer; MEDD, morphine equivalent daily dose; CYP-Opioids, Opioids subject to metabolism by CYP2D6; VAS, visual analogue scale; EQ, VAS, EuroQol Scale (0–100 mm); OWS, opiate withdrawal scale; GAF, global assessment of functioning; IQR, interquartile range, expressed in parenthesis as P25 and P75.

Opioid deprescription was effective in 76% of the cases with a 42% of opioid discontinuation after tapering without differences due to sex. Total median MEDD was 67% significantly reduced with a final consumption of 40 (0–80) mg/day [*p* < 0.001, difference Hodges-Lehmann: −80 (−83 to −40)]. In consonance, the percentage of patients with a high MEDD level (>100 mg/day) decreased significantly from 55% to 27% (*p* < 0.001) without differences due to sex. Interestingly, cases included in later time period (2016–2019) showed a significant lower final MEDD [0 (0–80) mg/day] compared to those included in early time-period (2013–2015) [60 (0–160) mg/day, *p* = 0.02] (Supplementary Table S1).

### 3.2 *CYP2D6* phenotype

Metabolic CYP2D6 phenotypes were classified as 6% PM, 85% EM and 9% UM according to their genotype without differences in frequency between sexes (females 6% PM, 84% EM and 78% UM) or compared with the control group (5% PM, 89% EM and 6% UM). Allelic frequencies of *CYP2D6* variants can be seen in Supplementary Table S2.

Here, UM phenotypes showed a significantly lower three-times MEDD compared to PM-EMs [40 (20–123) vs. 123 (80–226), *p* = 0.04, difference Hodges-Lehmann: −63 (−140 to 0)]. However, when only CYP2D6 metabolism mediated opioids were selected, no differences between CYP2D6 phenotypes and consumed MEDD were observed. What’s more, CYP2D6-UMs presented a lower rate of neuromodulators use in comparison to the other phenotypes in both basal and final visits (0% vs. 51%, *p* = 0.03 and 11% vs. 49%, *p* < 0.04, respectively) with no differences between sex or time period.

### 3.3 Opioid deprescription outcomes and *CYP2D6* phenotype

At final visit, even though a significant reduction in MEDD and opioid use was reached, most of the clinical outcomes remained stable without any significant changed after opioid deprescription or cessation. Only men showed a non-significant reduction of quality of life [basal vs. final, 49 (24) vs. 38 (23) mm, *p* = 0.05] while women remained stable [43 (24) vs. 46 (21) mm, *p* = 0.43].

**FIGURE 2:**
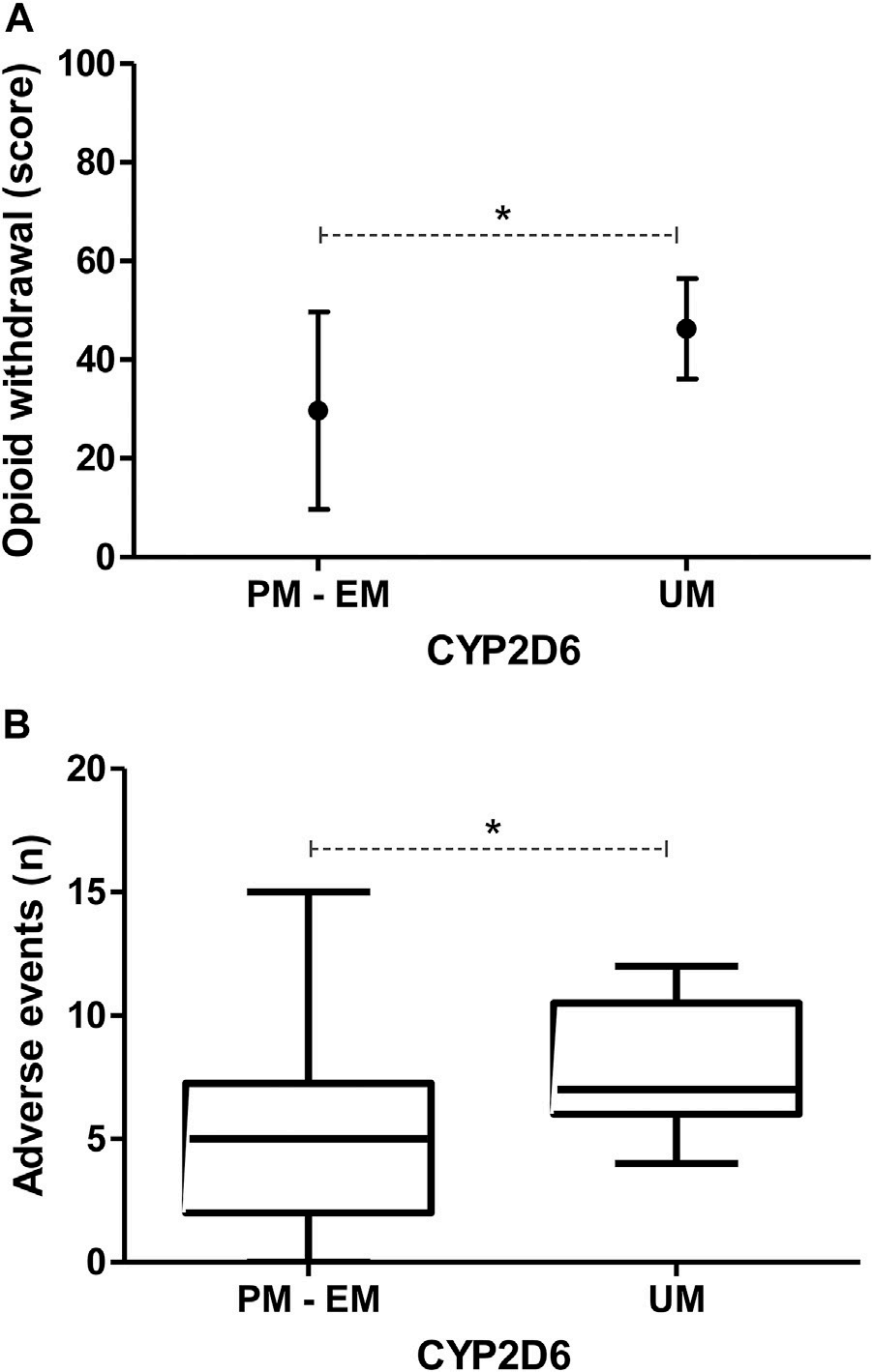
Final **(A)** opioid withdrawal scores (mean ± SD) and **(B)** total number of adverse events (boxplots) by *CYP2D6* metabolizer phenotype (PM, Poor Metabolizer; EM, Extensive Metabolizer; UM, Ultrarapid Metabolizer), showing a significant increase of both variables in UM. *p*-values <0.050 are represented with an asterisk.

Related to CYP2D6, UMs subjects (Figure 2A) showed a 3-4-fold increase in opioid withdrawal (46 (10) in comparison to the other phenotypes [30 (20) OWS scores, *p* = 0.01] with a significant inverse correlation with levels of quality of life, both in males [*r* = −0.572 (−0.797 to −0.209), *p* = 0.01] and females [*r* = −0.700 (−0.841 to −0.470), *p* < 0.001] (Supplementary Figure S1) at final visit. What´s more, PMs final functionality clearly improves to a mean of 90 GAF scores, which means “absent or minimal symptoms, good functioning in all areas, interested and involved in a wide range of activities. socially effective, generally satisfied with life, no more than everyday problems or concerns.” Whilst, UM decrease to 69 GAF scores, which means “some mild symptoms or difficulty in social, occupational, interpersonal relationships.”

### 3.4 Adverse events

A median of 5 (2–8) AEs per patient were reported in cases, being the most prevalent dry mouth, sleep disturbance, constipation and nervousness (present in >40% of the patients), while controls showed a lower frequency of AEs [3 (1–6) AEs/patient, *p* = 0.01] (Table 1). Cases included in 2016–2019 showed a significant lower frequency of AEs [6 (4–9) vs. 2 (0–5), *p* < 0.001] compared to those included earlier (2013–2015) (Supplementary Table S1). Furthermore, a total of 13% of the cases presented some suspected ADR (ratio 60 AEs: 1 ADR) during the deprescription, mainly psychiatric or reproductive system’s disorders.

Data related to AEs by CYP2D6 metabolic phenotype are shown in Table 3. Here, UMs showed a significantly higher mean of 7 (6–11) AEs/patient in comparison to the others phenotypes [5 (2–7) AEs/patient, *p* = 0.02], with higher frequencies of headache (100% vs. 33%, *p* = 0.01), edema (50% vs. 9%, *p* = 0.02), dry mouth (100% vs. 53%, *p* = 0.03) and nervousness (86% vs. 38%, *p* = 0.04) (Figure 2B and Supplementary Figure S2A). In accordance, UMs showed higher gastrointestinal (PM: 0 vs. EM: 71 vs. UM: 100, *p* = 0.01) and general (0% vs. 9% vs. 50%, *p* = 0.01) systems’ disorders. No gene-sex interactions by regression model were found in those variables where CYP2D6 metabolic phenotypes showed differences (data not shown).

**TABLE 3:** Adverse events frequency at basal and final visit and analysis by metabolic phenotype.

Adverse events (%)		*CYP2D6* phenotype				*p*-value^†^
	Visit	PM (*n* = 7, 6%)		EM (*n* = 98, 85%)	UM (*n* = 10, 9%)	
Total (Median (IQR))	*Basal*	4 (2–10)		6 (3–8)	7 (3–14)	0.57
	*Final*	2 (0–3)		5 (2–8)	**7 (6–11)** ^+^	**0.02**
Dry mouth	*Basal*	40		57	71	0.55
	*Final*	0		55	**100** ^+^	**0.01**
Sleep disturbing	*Basal*	40		52	71	0.52
	*Final*	33		55	71	0.52
Constipation	*Basal*	40		45	57	0.81
	*Final*	0		48	43	0.26
Nervousness	*Basal*	40		42	57	0.73
	*Final*	0		40	**86**	**0.02**
Dizziness	*Basal*	40		43*	43	0.99
	*Final*	0		23	43	0.32
Headache	*Basal*	60		26	57	0.09
	*Final*	33		33	**100** ^+^	**0.01**
Depression	*Basal*	40		34	43	0.88
	*Final*	0		39	75	0.05
Drowsiness	*Basal*	40		31	29	0.91
	*Final*	33		34	57	0.49
Weight change	*Basal*	0		35	43	0.24
	*Final*	0		28	17	0.49
Dry skin	*Basal*	20		31	43	0.70
	*Final*	0		38	67	0.14
Nausea	*Basal*	40		26	14	0.60
	*Final*	0		18	29	0.57
Itchy	*Basal*	40		25	43	0.49
	*Final*	0		27	43	0.37
Lack of appetite	*Basal*	20		28	43	0.65
	*Final*	0		23	57	0.09
Loss of libido	*Basal*	20		28	29	0.92
	*Final*	33		30	14	0.68
Vomiting	*Basal*	0		9	14	0.70
	*Final*	0		8	0	0.69
Edema	*Basal*	0		11	14	0.70
	*Final*	0		9	**50**	**0.01**
Skin redness	*Basal*	20		8	14	0.58
	*Final*	0		9	33	0.16
Sexual dysfunction	*Basal*	0		9	0	0.54
	*Final*	0		14	0	0.49

^†^ Comparisons between PM, vs. EM, vs. UM, for each visit were performed using ANOVA, or Kruskal-Wallis test for parametric and non-parametric data, respectively (significant *p* < 0.05 and highest value in bold). Multiple testing was adjusted with Bonferroni Correction where a *p*-value<0.017 was significant (+ highest value in bold and shaded in grey).

*p < 0.05 in basal vs. final (highest value in bold) using Fisher’s exact test. PM, poor metabolizer; EM, extensive metabolizer; UM, Ultrarapid Metabolizer. IQR, interquartile range, expressed in parenthesis as P25 and P75.

Related to sex, women reported a higher frequency of edema (15% vs. 0%, *p* = 0.05), dry mouth (63% vs. 33%, *p* = 0.02) and nervousness (50% vs. 22%, *p* = 0.029). Meanwhile, men retained sexual impotence issues at a significantly higher rate than females (25% vs. 4%, *p* = 0.01) mostly due to erectile dysfunction (Supplementary Figure S2B). What´s more, ADRs notified were three times higher in men than in women (23% vs. 7%, *p* = 0.02).

## 4 Discussion

Ambulatory opioid deprescription was effective in 76% of participants, where 42% ceased their opioid use. Here, CYP2D6-UMs showed the worst tolerability and high quality of life impact. Different frequencies of adverse events between sexes were reported that together with age and opioid dose could contribute to opioid dependence vulnerability.

This article also identifies priorities for monitoring younger, higher MEDD consumers with low tolerability CNCP patients who showed any misuse behavior. Current recommendations warn about a significant increase in OUD risk when the MEDD exceeds 90 mg/day (Busse et al., 2017; Webster, 2017). In our cases, a younger age and a higher median MEDD were found to be potential risk factors. Once OUD is detected, individualized decreasing dose regimen and/or opioid discontinuing is proposed based on clinical guidelines, which prevents the onset of withdrawal signs and symptoms (Nafziger and Barkin, 2018), as happened in our case. Additionally, our data demonstrates that UM phenotypes showed 3–4 times increased opioid withdrawal and higher AEs numbers that could be crucial at an early OUD stage (Planelles et al., 2019) or increasing the risk of life-threatening reactions compared to regular metabolizers (Haufroid and Hantson, 2015). In our setting, 42% completed the program without opioid prescription. Here, adherence monitored by qualitative urine drug testing and/or gas chromatography mass spectrometry as confirmatory quantitative testing could be considered (Nafziger and Barkin, 2018).

The study provides clear directions that would lead to changes in clinical practice. As a primary hypothesis, it was considered that CYP2D6-UM phenotypes patients with an OUD would show a different clinical outcome pattern when deprescribing, mainly due to a worse safety profile. The potential benefits of using CYP2D6 phenotype could be especially relevant in southern European and Northern African populations that have higher proportions of UM (Kirchheiner et al., 2008). In these situations, when PM or UM are detected, it is important to consider using different analgesic drugs, such as those which are metabolized through a phase II metabolic pathway, in order to avoid a possible therapeutic failure. Here, oxymorphone immediate- and/or sustained-release formulations could be considered in countries where they are available. For its part, tapentadol, while being residually metabolized to inactive hydroxytapentadol (2%) by CYP2D6, it is largely glucoronidated via phase II and interindividual CYP2D6-related variability in the analgesic response is not expected (Barbosa et al., 2016), which makes tapentadol an alternative to consider.

This study aims to demonstrate the clinical interest of genotyping when deprescribing in order to identify patients at risk of insufficient analgesia or adverse events. In this way, there is also a need to carry out studies that analyze the cost-effectiveness of genetic testing when genotyping is included in these procedures. Along with this, it is important the need to develop clinical guidelines as a vehicle to assist the providers of opioids, in order to detect a potential issue not only with *CYP2D6,* but also with other P450 enzymes (*1A2, 2C9, 2C19* or *B6*)*.*

Also, the need to implement pain research with a sex perspective is necessary to understand interindividual variability in terms of safety. Still, the remarkable female predominance in our study merits further attention. Nearly two thirds of our patients were adult women, given that female predominance in our CNCP population has been previously highlighted (Planelles et al., 2020). Furthermore, data showed that females communicated more AEs related to nervous, gastrointestinal and general systems, and less related to the sexual sphere in comparison to men, being third-less frequent ADRs in females (Muriel et al., 2019). Even more, surprisingly, men expressed a lower quality of life after opioid deprescription, while those of the women remained stable after deprescription. These different trends of impact related to the complex interdependence between biological sex and gender need to be elucidated (Becker and Chartoff, 2018; Rogers et al., 2020) because other factors (stress, depression, anxiety, responses to pain related to avoidance, coping) can have a greater impact on disability and quality of life, than on pain, *per se* (Sinha, 2008; Goodyear et al., 2018).

Some limitations should be taken under consideration. First, a convenience sample of patients attending a single pain clinic was established, along with this, a power analysis was not performed in order to know the best scenario to detect differences between groups. Furthermore, the total number of extreme phenotype subjects studied was relatively small. All this can compromise the power of statistical analyses, which may have made it difficult to detect significant differences between groups. Second, an 80% of UM were females, it would be difficult to assess the effect of CYP2D6 on the observed clinical outcome. Even more, drug inhibition or induction effects on CYP2D6 should be deeply analyzed (Kosten and Baxter, 2019), because it can condition the level of MEDD reduction (Smith et al., 2019). Furthermore, pharmacological data was obtained from EHRs and potential mismatches between the patients’ intake and prescribed doses could exist. Other drugs or interventions less commonly used in our setting such as tricyclic antidepressant, cannabinoid or nerves block should be explored in further analyses. Third, with basal and final visit data available, it is preferable to analyze the repeatedly measured data together instead of separate statistical tests, but the low frequency of extreme phenotype subjects limited its execution. Finally, since the inclusion period was long and substantial changes could have occurred, such as increased physician experience in deprescribing and/or new indications for available drugs, among others, subjects included in 2013–2015 and those in 2016–2019 were compared to determine if deprescription outcomes changed over time. Here, statistical significance was not reach for deprescription response, but lower MEDD (51% of the subjects ended with no opioids) combined with a welcome lower frequency of AEs were observed while clinical variables remained stable, strongly suggesting an improvement in the deprescription procedure over time.

In conclusion, CYP2D6 metabolizer phenotypes may contribute to differential and improved opioid deprescription in CNCP. Sex may play a relevant role in the tolerability when deprescribing. Further studies considering these potential genetics, as well as sex/gender differences could help to understand the interindividual variability in real-world patients.

## Data Availability

The raw data supporting the conclusion of this article will be made available by the authors, without undue reservation.

## Supplementary material

SUPPLEMENTARY FIGURE S1 Opioid withdrawal and quality of life inverse correlation after deprescription programme in OUD males and females.

SUPPLEMENTARY FIGURE S2 Percentage of Adverse Events at final visit classified by: **(A)** CYP2D6 metabolic phenotype (PM, Poor Metabolizer; EM, Extensive Metabolizer; UM, Ultrarapid Metabolizer); **(B)** Sex-differences (F, female, M, male). *p-*values <0.050 are represented with an asterisk.
