# Supplementary figures and images for: Impact of CYP2D6 genotype on opioid use disorder deprescription: an observational prospective study in chronic pain with sex-differences
Source: Front Pharmacol. 2023 May 31;14:1200430. doi: 10.3389/fphar.2023.1200430 (PMC10264765; doi:10.3389/fphar.2023.1200430)

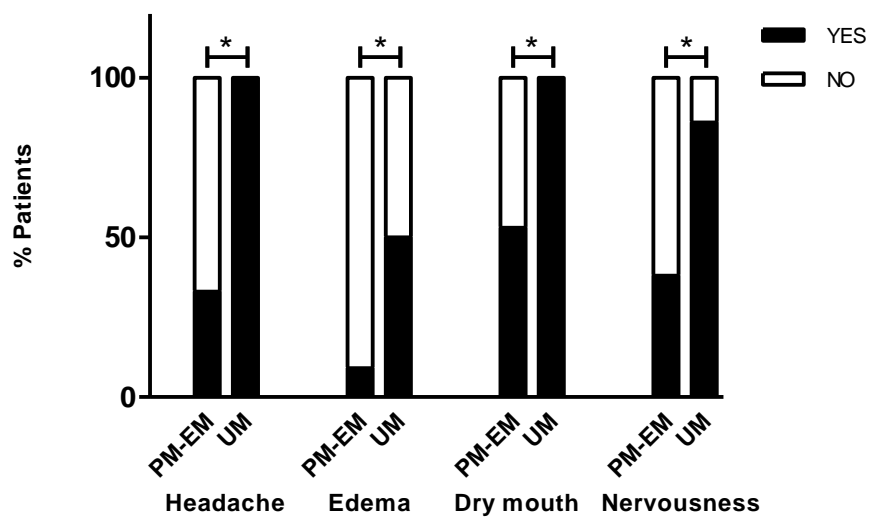

Supplement: Supplementary file 1 [file DataSheet2.PDF]

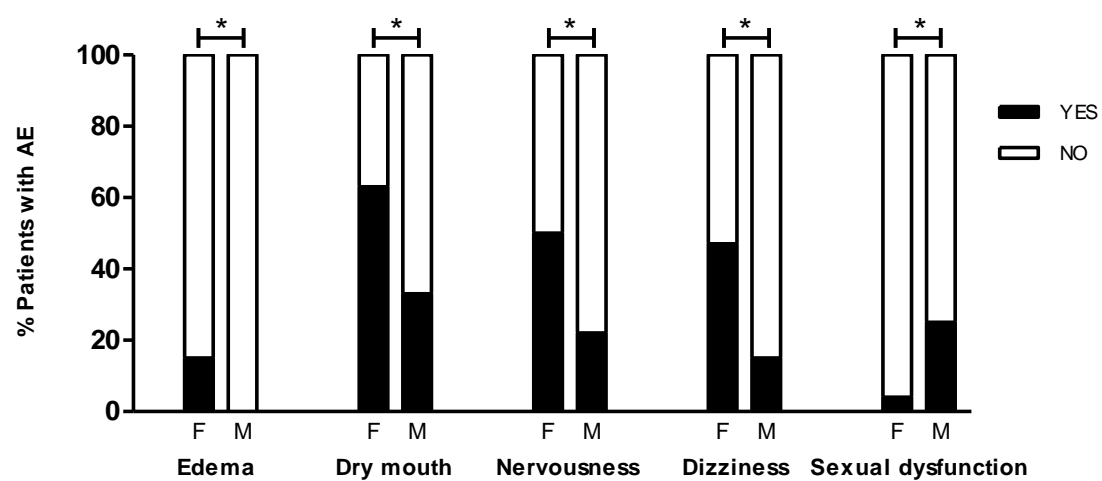

Supplement: Supplementary file 3 [file DataSheet3.PDF]

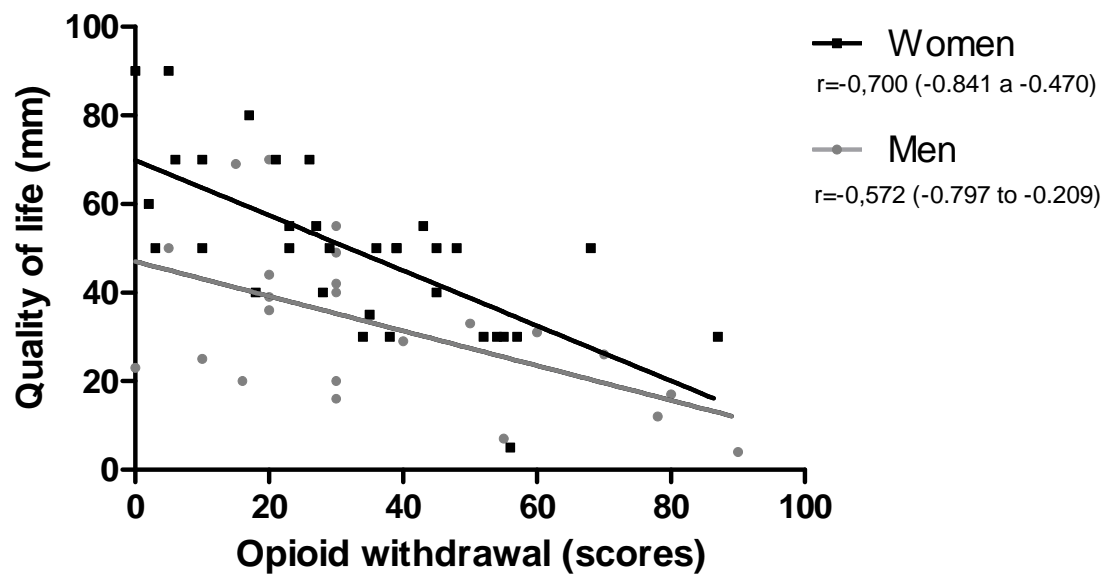

Supplement: Supplementary file 5 [file DataSheet1.PDF]

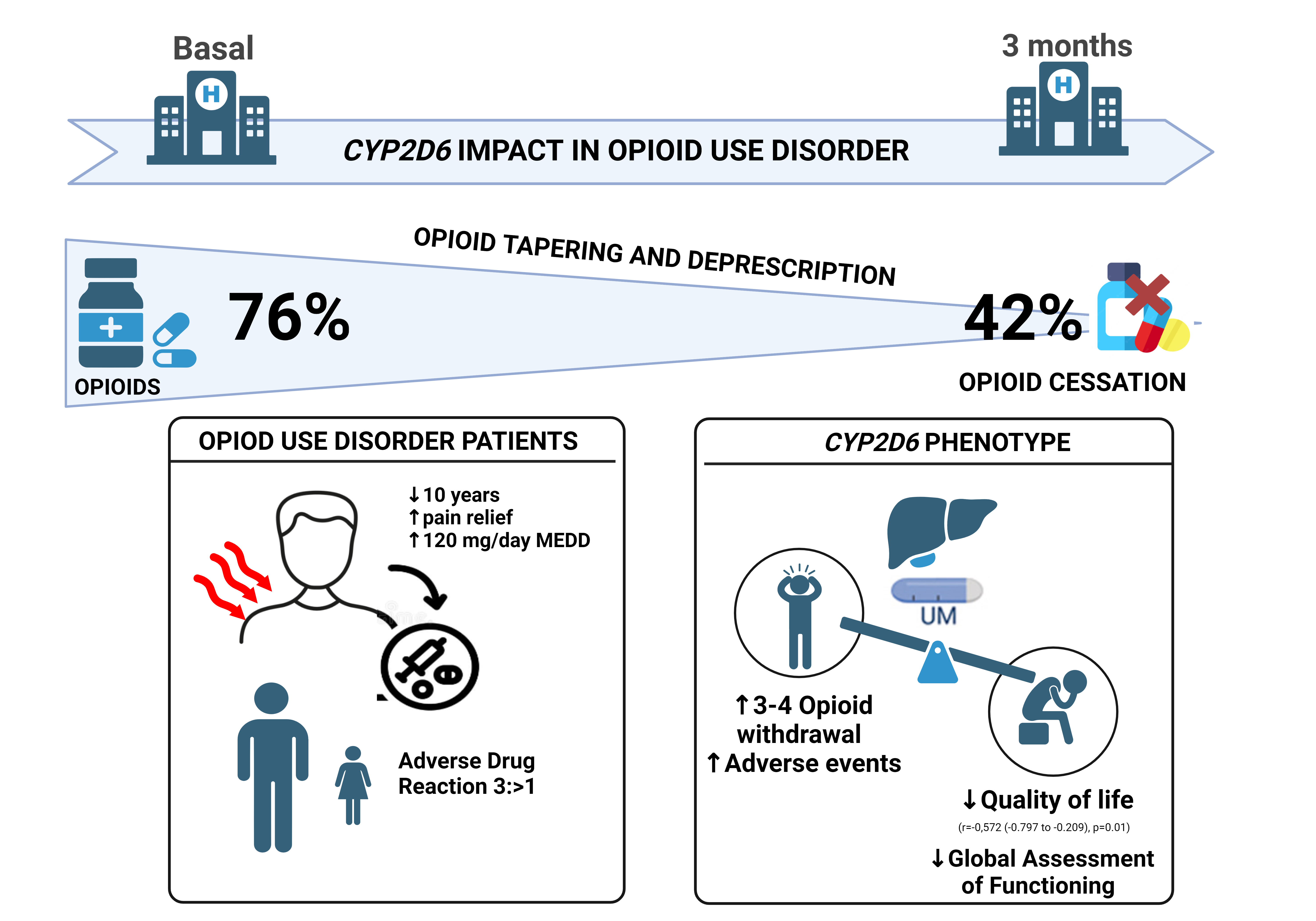

Supplement: Supplementary file 6 [file Image1.jpg]
