# Supplementary material for: Impact of CYP2D6 genotype on opioid use disorder deprescription: an observational prospective study in chronic pain with sex-differences
Source: Front Pharmacol. 2023 May 31;14:1200430. doi: 10.3389/fphar.2023.1200430 (PMC10264765; doi:10.3389/fphar.2023.1200430)
Supplement: Supplementary file 2 [file Table1.DOCX]

**Supplementary Table 1.** Sociodemographic, clinical, pharmacological and tolerability variables in subjects included in 2013-2015 and in 2016-2019 at their final visit after deprescription.

| **Mean (SD)** | **2013-2015**  (n=88) | **2016-2019**  (n=50) | **p-valor^†^** |
| --- | --- | --- | --- |
| **Age** (years) | 53 (12) | 57 (13) | 0.05 |
| **Sex** (female, %) | 64 | 70 | 0.55 |
| **Deprescription Responder** (%) | 70 | 86 | 0.07 |
| **Final opioid use** (%) | 63 | 49 | 0.14 |
| **Final MEDD**  (mg/day, median (IQR)) | **60 (0-160)*** | 0 (0-80) | **0.02** |
| **MEDD >100 mg/day** (%) | 31 | 21 | 0.29 |
| **Pain Intensity**  (VAS, 0-100 mm) | 55 (29) | 66 (24) | 0.05 |
| **Pain Relief**  (VAS, 0-100 mm) | 40 (30) | 39 (25) | 0.91 |
| **Quality of life**  (EQ, 0-100 mm) | 46 (23) | 38 (20) | 0.06 |
| **Opioid Withdrawal**  (OWS, 0 - 96 score) | 33 (21) | 31 (16) | 0.81 |
| **Functionality**  (GAF, 0-100 score) | 69 (14) | 71 (29) | 0.83 |
| **AEs** (median (IQR)) | **6 (4-9)*** | 2 (0-5) | **<0.001** |

^†^ Group comparisons using t-test and U Mann-Whitney test for continuous parametric and non-parametric data, repectively, and Ficher’s exact test for categorical data (significant p<0.05 in bold)

VAS: Visual Analogue Scale; EQ: VAS EuroQol Scale (0-100 mm); AEs: adverse events; IQR: Interquartile range.

* p<0.05 (highest value in bold)
