# Supplementary material for: Impact of CYP2D6 genotype on opioid use disorder deprescription: an observational prospective study in chronic pain with sex-differences
Source: Front Pharmacol. 2023 May 31;14:1200430. doi: 10.3389/fphar.2023.1200430 (PMC10264765; doi:10.3389/fphar.2023.1200430)
Supplement: Supplementary file 4 [file Table2.DOCX]

**Supplementary Table 2.** Allelic frequencies of *CYP2D6* polymorphisms detected in our study.

| **Genetic variant** | **Genotype** (%) | | **Allele Frequency** | |
| --- | --- | --- | --- | --- |
| *CYP2D6*2*  (1584 C>G) | C/C | 57 | C | 0.72 |
|  | C/G | 30 |  |  |
|  |  |  | G | 0.28 |
|  | G/G | 13 |  |  |
| *CYP2D6*4*  (1846 G>A) | G/G | 82 | G | 0.88 |
|  | G/A | 12 |  |  |
|  |  |  | A | 0.12 |
|  | A/A | 6 |  |  |
| *CYP2D6*10*  (100 C>T) | C/C | 64 | C | 0.80 |
|  | C/T | 27 |  |  |
|  |  |  | T | 0.20 |
|  | T/T | 9 |  |  |
| *CYP2D6*3*  (2549Del>A) | A/A | 99 | A | 0.99 |
|  | A/Del | 1 |  |  |
|  |  |  | Del | 0.01 |
|  | Del/Del | 0 |  |  |
| *CYP2D6*6*  (1707 T>Del) | T/T | 99 | T | 0.99 |
|  | T/Del | 1 |  |  |
|  |  |  | Del | 0.01 |
|  | Del/Del | 0 |  |  |
| *CYP2D6*35*  (31G>A) | G/G | 89 | G | 0.94 |
|  | G/A | 11 |  |  |
|  |  |  | A | 0.06 |
|  | A/A | 0 |  |  |
| *CYP2D6*41*  (2988G>A) | G/G | 84 | G | 0.92 |
|  | G/A | 15 |  |  |
|  |  |  | A | 0.08 |
|  | A/A | 1 |  |  |
| *CYP2D6*17*  (1023C>T) | C/C | 97 | C | 0.99 |
|  | C/T | 3 |  |  |
|  |  |  | T | 0.01 |
|  | T/T | 0 |  |  |
| *CYP2D6*29*  (3183 G>A) | G/G | 100 | G | 1.00 |
|  | G/A | 0 |  |  |
|  |  |  | A | 0.00 |
|  | A/A | 0 |  |  |
